# Pro-tumorigenic role of type 2 diabetes-induced cellular senescence in colorectal cancer

**DOI:** 10.3389/fonc.2022.975644

**Published:** 2022-08-18

**Authors:** Francesco Melia, Palita Udomjarumanee, Dmitry Zinovkin, Nahid Arghiani, Md Zahidul Islam Pranjol

**Affiliations:** ^1^ Department of Biochemistry and Biomedicine, School of Life Sciences, University of Sussex, Brighton, United Kingdom; ^2^ Department of Immunology and Inflammation, Faculty of Medicine, Imperial College London, London, United Kingdom; ^3^ Department of Pathology, Gomel State Medical University, Gomel, Belarus; ^4^ Department of Molecular Biosciences, the Wenner-Gren Institute, Stockholm University, Stockholm, Sweden

**Keywords:** senescence, diabetes, colon cancer, fibroblast, endothelial cells, tumour microenvironment

## Abstract

Colorectal cancer (CRC) is the second leading cause of cancer-related mortality worldwide. The disease still remains incurable and highly lethal in the advanced stage, representing a global health concern. Therefore, it is essential to understand the causes and risk factors leading to its development. Because age-related cellular senescence and type 2 diabetes (T2D) have been recognised as risk factors for CRC development, the recent finding that type 2 diabetic patients present an elevated circulating volume of senescent cells raises the question whether type 2 diabetes facilitates the process of CRC tumorigenesis by inducing premature cell senescence. In this review, we will discuss the mechanisms according to which T2D induces cellular senescence and the role of type 2 diabetes-induced cellular senescence in the pathogenesis and progression of colorectal cancer. Lastly, we will explore the current therapeutic approaches and challenges in targeting senescence.

## Introduction

Colorectal cancer (CRC) is the second leading cause of cancer-related mortality worldwide and the third most common type of cancer (1). In 2020, global CRC cases increased by 6%, with a mortality rate of 29 per 100,000 people in men and 20 per 100,000 people in women (1). In the UK, there are around 42,900 new colorectal cancer cases every year (2). However, CRC incidence and mortality rates have decreased by 6% and 12% respectively. In regard to survival, around 52.9% of patients diagnosed with CRC survive the disease for ten years or more (2). Despite the decline in CRC incidence and mortality over the last decade, mainly attributed to the improvement in early screening methods, CRC still remains incurable and highly lethal in the advanced stage (3). Because of its magnitude, CRC represents a global health concern and therefore it is essential to understand the causes and risk factors leading to its development.

Several risk factors and pathologies, including ageing, smoking, obesity and diabetes have been associated with poor prognosis in colorectal cancer patients. Ageing represents the major risk factor for CRC development. Brenner et al. (2007) showed that the transition rates from advanced adenoma to CRC strongly increase with age, from 2.6% in age groups 55-59 years to 5.6% in age group ≥ 80 years among women, and from 2.6% in age group 55–59 years to 5.1% in age group ≥ 80 years among men (4). In addition, Siegel et al. (2020) showed that CRC incidence rates increase by 30% every 5 years of age in individuals aged 50 and over (5). The ageing process is thought to contribute to tumorigenesis *via* aberrant genome maintenance and systemic inflammation that result in tissue damage and occurrence of unfavourable genome modifications (6). More recently, cellular senescence has been considered as an additional cause of age-related tumorigenesis. Senescence is a stress-response cellular state characterised by proliferative arrest but active metabolism (7). Over lifetime, due to the action of several stressors such as DNA damage and telomere shortening, senescent cells accumulate in the organism and release a variety of pro-inflammatory cytokines responsible for low-grade inflammation.

This age-related inflammation, also referred to as inflammaging, increases the risk for tissue damage and genetic aberrations that cause cellular transformation and cancer development (8, 9). However, cellular senescence is not exclusive to ageing. Age-related and metabolic diseases such as type 2 diabetes (T2D) represent a source of cellular stress due to their disruptive effect on normal physiological processes and, therefore, can induce premature senescence (10). In fact, several studies have shown that T2D induces senescence in multiple types of cells, including fibroblasts and endothelial cells (11, 12). T2D has also been recognised as a risk factor for CRC development. For example, Xiao et al. (2022) showed that diabetes was associated with increased risk of both right-sided colorectal cancer (Relative risk [RR] = 1.35, 95% CI = 1.24 - 1.47) and left-sided colorectal cancer (RR = 1.18, 95% CI = 1.08 - 1.28) using data regarding 1,642,823 individuals and 17,624 colon cancer patients (13). Ma et al. (2018) also suggested that T2D is associated with increased risk of CRC development (Hazard ratio [HR]: 1.42; 95% CI: 1.12 - 1.81) (14). The peculiar association between CRC, senescence and T2D raises the question whether the T2D-induced premature senescence facilitates the process of CRC tumorigenesis in T2D patients (15–17).

In addition, the recent finding that T2D patients present a higher circulating volume of senescent T cells compared to their age-matched healthy counterparts supports the hypothesis that T2D pathophysiology is also implicated in premature immunosenescence (18). T-cells also play a key role in immune control of the colorectal carcinoma microenvironment (19). Considering the importance of T cells in the response against cancer, T cell senescence would be a detrimental factor for the organisms because of its tumorigenic potential as well as reduced anti-cancer response. The distinctive association between CRC, senescence and T2D is intricate due to several aspects such as the anatomical location and complexity of the tumour microenvironment. However, the lack of early CRC diagnosis reflects the partial understanding of some of the processes leading to the disease (20). Thus, cellular senescence could represent a novel mechanism. Despite the supporting evidence, the correlation between CRC, senescence and T2D still remains unclear. In this review, we will discuss the mechanisms according to which T2D induces cellular senescence, and the potential role of T2D-induced senescence in the development of colorectal cancer. Particularly, we will focus on the tumorigenic activity of senescent fibroblasts, endothelial cells and T cells within the tumour microenvironment. Lastly, we will explore the current therapeutic challenges, approaches and future perspectives in targeting senescence.

## Cellular senescence

Cellular senescence is a stress-response process characterised by changes in gene expression that ultimately lead to the alteration of cellular phenotype and metabolism (7). The concept of cellular senescence was discovered approximately 60 years ago by Hayflick and Moorhead (1961) but its roles in physiological processes and diseases have recently emerged (21, 22).

Senescent cells are denoted by proliferative arrest, alteration of morphology and secretome, and resistance to apoptosis (7, 23). The arrest in cell division, which is the major hallmark of senescence, prevents the progression of damaged cells into malignancy (7). The altered secretome results in the secretion of pro-inflammatory cytokines, proteases and growth factors that are collectively referred to as the senescence-associated secretory phenotype (SASP). SASP exerts paracrine action on the surrounding environment and is involved in the attraction of immune cells, stimulation of angiogenesis and cell proliferation in a process that mimics a wound healing response. Consequently, cellular senescence is a key mechanism in wound healing and tissue repair (23–26). Most senescent cells also express high levels of the enzyme β-galactosidase at pH 6. This enzymatic activity, initially described by Dimri et al. (1995), enabled identification of senescent fibroblasts and keratinocytes in biopsies of aged human skin, and subsequently the enzyme became known as senescence-associated β-galactosidase (SA-β-Gal) (27).

The stressors responsible for the induction of cellular senescence are categorised into acute and chronic stressors, each determining a different outcome: acute stressors stimulate tissue repair and wound-healing response whereas chronic stressors can induce persistent senescence activation and accumulation of senescent cells, leading to continuous SASP release. As a result, chronic SASP generates low-grade inflammation, excessive cell proliferation and angiogenesis, causing tissue damage and potentially contributing to the promotion of a pro-tumorigenic environment (8, 23, 28, 29). Therefore, whilst acute senescence seems to be a programmed process, the switch from a temporary to a persistent senescent state appears to be unscheduled in nature (8). Some of the cellular stressors are endogenous and part of the normal cell cycle such as telomere shortening during DNA replication, DNA damage and reactive oxygen species (ROS) produced by mitochondria during normal metabolism (7, 30, 31). These events arise with age and so does cellular senescence. Other stressors are environmental factors, obesity, and ongoing pathologies such as T2D and hypertension (32–35).

In the context of cancer, the senescence-induced proliferative arrest is an important tumour-suppressive mechanism. However, the SASP released by senescent cells possesses both pro- and anti-tumorigenic abilities on the surrounding environment: acute senescent cells present anti-tumorigenic potential because the pro-inflammatory component of their SASP is associated with the recruitment of immune cells at the site of the tumour, therefore promoting a tumour-specific immune response. However, acute SASP action is limited and should be considered as an anti-cancer mechanism against pre-tumorigenic cells rather than malignant cells. On the other hand, chronic senescent cells do not present anti-tumorigenic properties but can instead contribute to the generation of a pro-tumorigenic environment due to the prolonged inflammatory state caused by their SASP (9, 29, 36, 37).

Despite the common characteristics, the phenotype of senescent cells vary depending on the cell type and senescence-inducing stressor (38). For example, senescent T cells are characterised by specific surface markers that allow to detect their differentiation and senescent state. These cells lack CD27 and CD28 surface markers but express markers such as CD57 and KLRG-1 which determine a decrease in cellular proliferation (39–41). Among senescent T cells there are the effector memory T cells re-expressing the surface receptor CD45RA, referred to as EMRA T cells. EMRA T cells have reduced proliferative capacity and display SASP secretion (42, 43). However, whether these cells can be considered fully senescent cells or not is still under debate (44, 45).

## Type 2 diabetes induces premature cellular senescence

Chronic hyperglycaemia is the major contributing factor to T2D-associated cardiovascular complications such as retinopathy, nephropathy and hypertension (46). Along with these complications, hyperglycaemia has been shown to induce cellular senescence in fibroblasts, endothelial cells and more recently in mesenchymal stem cells such as umbilical cord-derived mesenchymal stem cells (11, 12, 47). These senescent stem cells are characterised by multipotentiality loss in addition to the hallmarks of senescence (47). Here, we propose a general mechanism according to which hyperglycaemia activates senescence-inducing pathways.

**Figure 1:**
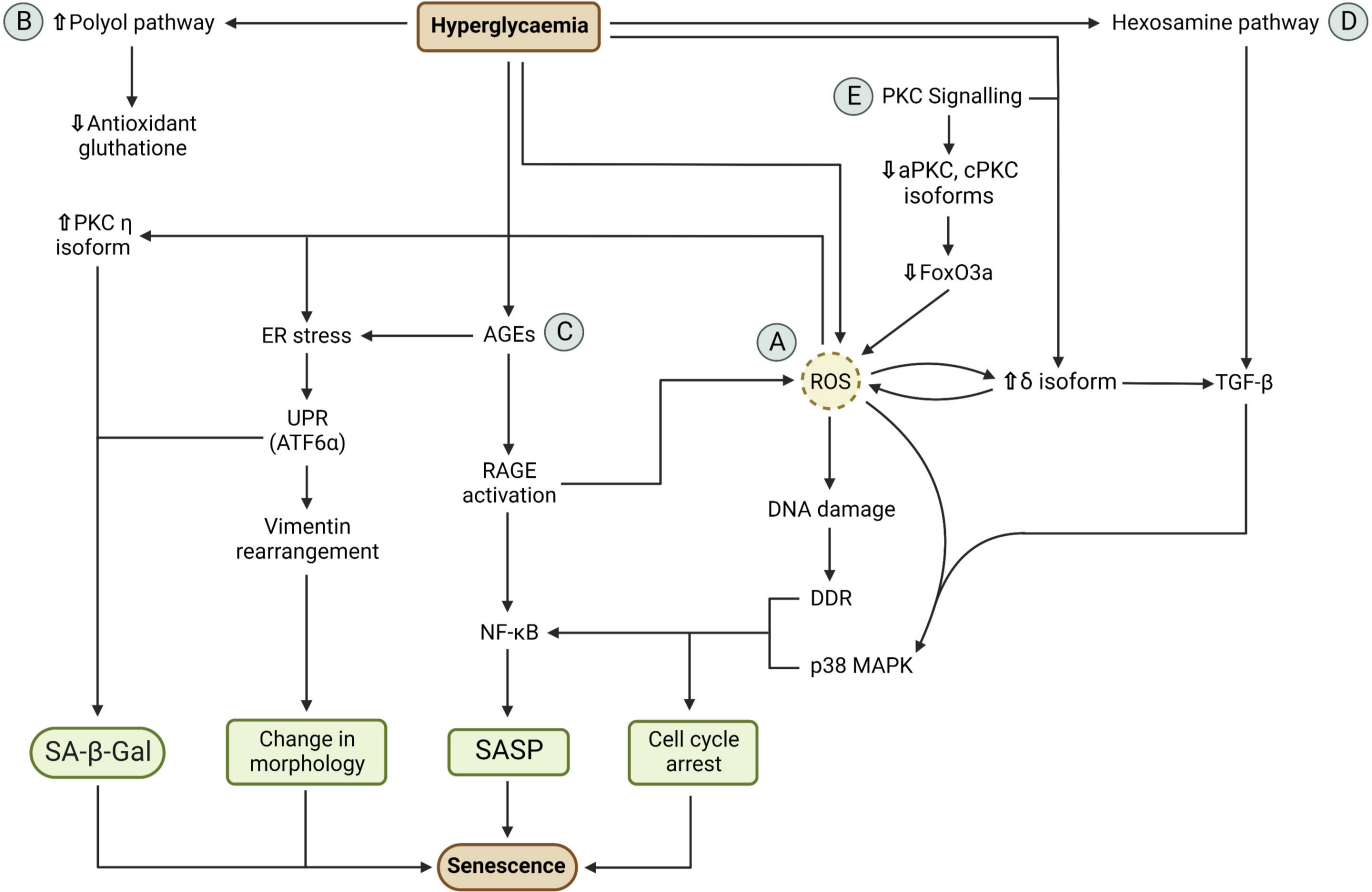
Mechanism of hyperglycaemia-induced cellular senescence. **(A)** Hyperglycaemia induces ROS overproduction *via* mitochondria overload which results in oxidative stress. ROS causes DNA damage response (DDR) activation, due to DNA oxidative damage, and p38 MAPK pathway activation. DDR and p38 MAPK determines cell cycle arrest and NF-κB upregulation. NF-κB activation results in SASP secretion. ROS also generates ER stress *via* chemical modification of ER proteins. ER stress activates the unfolded protein response (UPR). The activation of the ATF6α branch of the UPR causes expression of SA-β-Gal and changes in cellular morphology *via* cytoskeletal vimentin rearrangement. **(B)** Hyperglycaemia increases polyol pathway activity, causing reduced antioxidant glutathione synthesis due to reduce NADPH availability. Glutathione deficiency contributes to the inability of the cell to counteract oxidative stress. **(C)** Hyperglycaemia causes advance glycation end products (AGEs) *via* glycation of intracellular and extracellular proteins. Intracellular AGEs cause ER stress which results in SA-β-Gal activity and change in cellular morphology. Extracellular ages cause AGE receptor (RAGE) activation which results in ROS production and NF-κB activation. This ultimately results in cell cycle arrest and SASP secretion. **(D)** Hyperglycaemia results in increased hexosamine pathway activity due to increased glucose-6-phosphate production. This pathway produces N-acetyl glucosamine (GlcNAc) which induces TGF-β expression. TGF-β activates the p38 MAPK which results in cell cycle arrest and SASP secretion. **(E)** PKC signalling contributes to the activation of senescence pathways. Hyperglycaemia results in increased diacyl glycerol production and activation of PKC δ isoform. PKC δ activation causes TGF-β expression and ROS production which, in turn, activate PKC δ in a positive feedback loop mechanism. In addition, ROS also activate PKC η which induces SA-β-Gal activity. Downregulation of aPKC and cPKC results in inactivation of FoxO3a which results in ROS production. Cell cycle arrest, SASP secretion, change in cellular morphology and SA-β-Gal activity are the major characteristics of senescence.

Intracellular hyperglycaemia induces oxidative stress, proteostasis alteration and dysregulation of protein kinase C (PKC) signalling (48–54) (**Figure 1**). These pathways are integrated and promote the establishment of the major characteristics of senescence: cell cycle arrest, changes in cellular morphology, SASP secretion and SA-β-Gal activity. It should be emphasised that these pathways are mechanistically similar in both age-related and T2D-induced cellular senescence, but the inducing stimuli and time span are different: during ageing, senescence-inducing stimuli such as telomere erosion occur over a long period of time while in T2D senescence is accelerated by stimuli such as hyperglycaemia and hypertension (55, 56). A pivotal pathway involved in senescence activation is the p38 mitogen-activated protein kinases (MAPK) pathway. The p38 MAPK is a stress-response pathway activated by several stressors among which are ROS and transforming growth factor β (TGF-β) (54, 57). P38 activation leads to cell cycle arrest and SASP secretion whereas endoplasmic reticulum (ER) stress contributes to the changes in cellular morphology and expression of SA-β-Gal (58–60). However, the process that leads to intracellular hyperglycaemia is still not fully understood. For example, activated T cells express insulin receptors and, unlike naive T cells, insulin signalling increases GLUTs expression on the plasma membrane (61, 62). As a result, the T2D hyperinsulinemic environment may induce GLUT transporters overexpression, leading to intracellular glucose concentration rising in parallel with serum hyperglycaemia (48). By contrast, other studies have suggested that vascular endothelial and smooth muscle cells downregulate GLUTs expression in response to hyperglycaemia (63, 64).

### Oxidative stress

As a part of their normal metabolic activity, mitochondria produce ROS and reactive nitrogen species by-products *via* the complexes of the electron transport chain (30). In a hyperglycaemic environment, the excessive glucose oxidation causes mitochondrial overload, resulting in ROS overproduction and increased ATP/ADP ratio (51, 53). In addition, intracellular hyperglycaemia promotes polyol pathway activation. In this pathway, the enzyme aldose reductase converts glucose to sorbitol, which is then oxidised to fructose. However, in the conversion from glucose to sorbitol, aldose reductase consumes NADPH which is also a cofactor used for the generation of the antioxidant glutathione (49). Therefore, high glucose concentration shifts the equilibrium towards the polyol pathway, resulting in reduced glutathione synthesis. The reduction in this antioxidant levels reduces the ability of the cell to counteract ROS overproduction as well as other free radicals.

The oxidative stress caused by this ROS imbalance and reduced antioxidant determines mitochondrial DNA oxidative damage, leading to mitochondrial dysfunction and, as a consequence, impairment of cellular metabolism (50, 65). Oxidative stress also causes nuclear DNA oxidative damage and activation of the DNA-damage response (DDR) pathway. DDR results in cell cycle arrest *via* the p16^INK4a^/Rb pathway and upregulation of the CDK-inhibitor p21 (66–69). DDR is also involved in the generation of SASP *via* the activation of the kinases ataxia-telangiectasia mutated (ATM) and ATM- and Rad3-Related (ATR) which belong to the phosphatidylinositol-3 kinase-related kinases (PIKK) family (70). ATM and ATR have been shown to activate the transcription factor GATA4, a novel positive regulator of senescence that activates NF-kB, ultimately leading to SASP formation (71).

Increased cellular ROS also activate the p38 MAPK pathway. Similarly to the DDR, p38 MAPK activation results in cell cycle arrest *via* p16^INK4a^/Rb and p21 pathways (59). In addition, p38 MAPK induces SASP production through NF-kB activation (57, 69). Conversely, p38 MAPK inhibition by the selective inhibitor SB203580 effectively collapses the senescence-associated cytokine network, preventing the SASP paracrine effects of senescent cells (69). The p38 MAPK pathway can also be activated by intracellular hyperglycaemia *via* the hexosamine pathway. In fact, high intracellular glucose levels determine increased levels of the glycolytic intermediate fructose-6-phosphate (F6P). Part of this sugar is used in the hexosamine pathway. In this pathway, F6P is converted into uridine diphosphate (UDP) and N-acetyl glucosamine (GlcNAc). The latter can interact with transcription factors that induce the expression of TGF-β1 which, in turn, activates the p38 MAPK pathway (49, 72).

### Proteostasis alteration

Proteostasis can be defined as the ability of the cell to maintain a functional cellular proteome (50). Loss of proteostasis due to protein misfolding has detrimental effects on cellular function. One of the causes of such event is a non-programmed chemical modification, such as glycation or carbonylation, of cellular proteins (50).

Glycation is a non-enzymatic reaction of glucose with proteins that leads to the formation of molecular products known as advanced glycation end products (AGEs) involved in diseases and ageing (48). AGEs are formed both intracellularly and extracellularly and alter cellular proteostasis in T2D. After cytoplasmic glucose-6-phosphate (G6P) is transported into the ER *via* the transporter G6PT, glucose then glycates the proteins present in the ER lumen, causing protein misfolding and ER stress. ER stress results in the activation of the unfolded protein response (UPR) pathway (65, 68, 73, 74). Extracellular AGEs precursors interact and modify connective tissue components, such as collagen and plasma proteins, and bind to the AGE receptor (RAGE) present on the cell surface (75, 76). RAGE activation has been shown to activate the transcription factor NF-kB by degradation of the IkB proteins and to induce the production of cytosolic ROS (75, 77).

Carbonylation is an irreversible reaction of ROS with proteins. Carbonylation of ER enzymes involved in protein folding, such as protein disulphide isomerase (PDI) and calreticulin proteins, causes proteins misfolding and aggregation into structures known as lipofuscin that are resistant to proteolytic degradation (50, 78). These events cause accumulation of misfolded proteins and ER stress, resulting in the activation of UPR. The ATF6α branch of the UPR pathway has been shown to be involved in cellular senescence by increasing SA-β-Gal activity and by altering the cellular morphology *via* changes in cytoskeletal vimentin (60). It should be noted that ER stress involves complex signalling pathways and the cell may reinstate its normal proliferative activity by resolving the proteostasis alteration rather than activating senescence-inducing pathways (79). However, it remains unclear whether UPR downstream signalling results in either proliferation or senescence as this depends on the nature and intensity of the stimuli involved (79). It is plausible that UPR senescence-inducing pathways arise from stimuli that cause sustained damage which is, however, insufficient to trigger apoptosis.

### PKC signalling

Activation or downregulation of certain PKC isoforms has been shown to promote senescence. Hyperglycaemia determines an increase in diacyl glycerol (DAG) production, which is responsible for the activation of DAG-sensitive PKC isoforms such as cPKC, PKC δ and PKC η (54, 80). PKC δ activation promotes expression of TGF-β and induces ROS production which, in turn, activates PKC δ in a positive feedback loop mechanism (54, 81). PKC η activation has been shown to induce SA-β-Gal expression. For instance, PKC η-knockdown MCF-7 cells showed SA-β-Gal expression reduced by 2-fold compared to the scrambled controls (p ≤ 0.0001). Importantly, PKC η is also activated by ROS (82). On the other hand, aPKC or cPKC downregulation also promotes senescence. The downregulation of these PKC isoforms has been shown to inhibit the nuclear import of the transcription factor FoxO3a *via* AKT-mediated phosphorylation. Specifically, aPKC or cPKC inhibition in HCT116 cells resulted in a 10-fold increase in SA-β-Gal expression compared to the control (p < 0.05). In these cells, the levels of phosphorylated FoxO3a were increased by two-fold compared to the controls (p < 0.05) (80). FoxO3a inactivation has been shown to induce ROS production, therefore contributing to cellular oxidative stress (83).

## Senescent cells modulate the tumour microenvironment through SASP release

The tumour microenvironment (TME) is a complex network of cellular and molecular components consisting mainly of tumour-infiltrating cells, blood vessels, extracellular matrix (ECM) and other matrix-associated molecules (84). Persistent inflammation in the TME has been recognised as a tumour initiator and promoter because it serves as a chemoattractant for the recruitment of tumour-infiltrating cells that support tumour growth and metastasis (85). Consequently, the chronic systemic inflammation that characterises T2D represents a risk factor for cancer development (16). T2D interferes with the normal colon tissue physiology in multiple processes. Firstly, AGEs accumulation and TGF-β upregulation determine ECM accumulation, cross-linking of collagen, thickening of basement membrane, loss of elasticity and fibrosis [76]. These events result in colon wall remodelling and change in its biomechanical properties (86, 87). Secondly, increased levels of insulin determine activation of the insulin-like growth factor 1 (IGF-1) receptor, which stimulates cell growth and proliferation (88). For instance, Teng et al. (2016) showed that the treatment of MC38 cells with 50 ng/mL of insulin and 50 ng/mL of IGF-1 determined a 2-fold increase in cell proliferation compared to the negative control (p < 0.001). In mouse models, the tumour growth of MC38 cells was doubled in mice that had serum insulin and serum IGF-1 6 times and 5 times higher than the control, respectively (p < 0.001) (88). Therefore, the extracellular matrix remodelling and the proliferative stimulation *via* IGF-1 activation may alter the colon tissue into a pro-tumorigenic environment (89). An additional and less explored role of T2D on CRC tumorigenesis is represented by T2D-induced cellular senescence. Chronic senescent cells may have the ability to modulate and enhance the inflammatory state of the tumour microenvironment *via* the release of pro-inflammatory SASP. In support to this hypothesis, multiple studies demonstrated the ability of senescent fibroblasts, endothelial cells and T cell to induce tumour growth and metastasis.

### Fibroblasts

**Figure 2:**
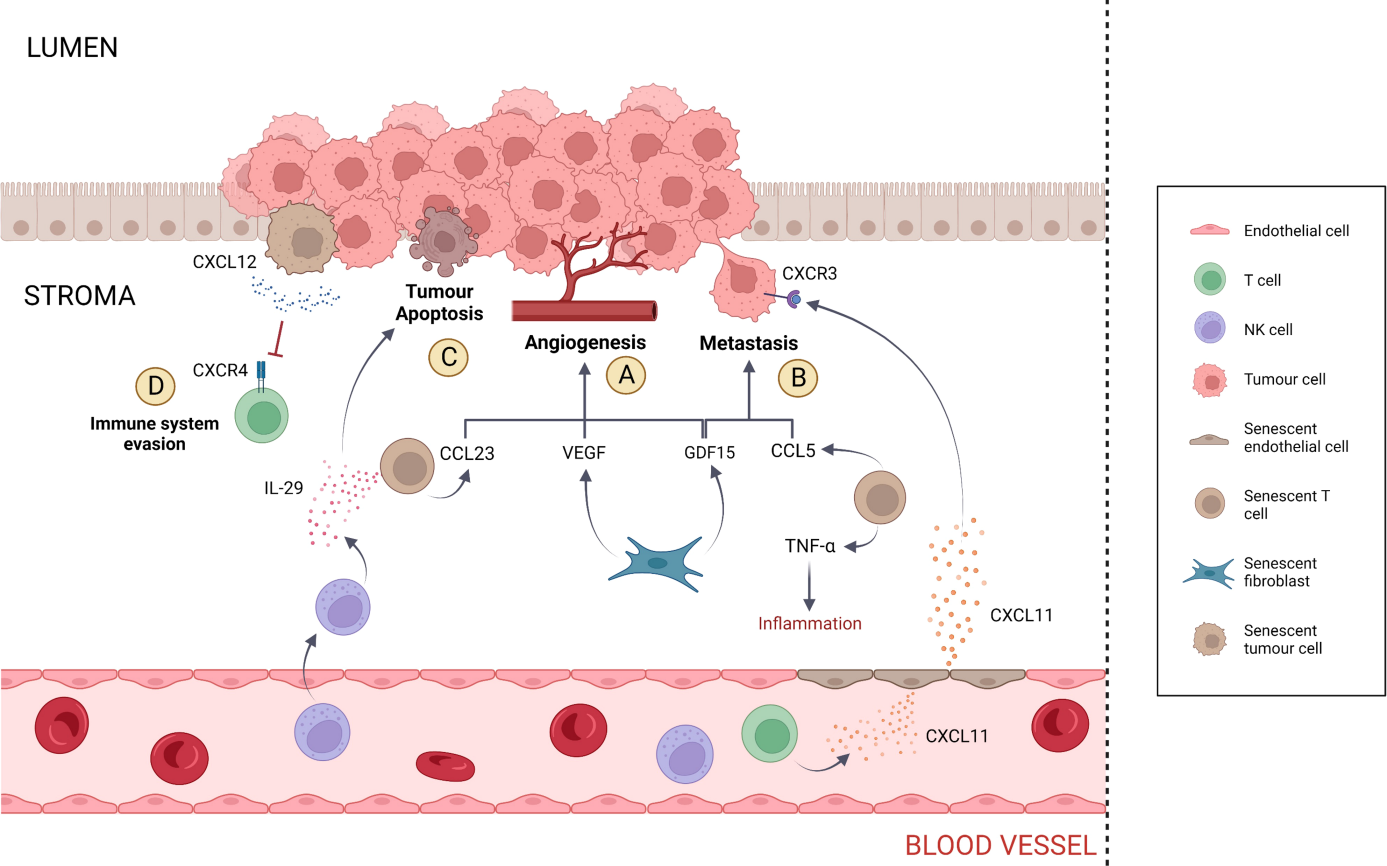
SASP activity within the tumour microenvironment. **(A)** Angiogenesis is stimulated by CCL23, VEGF and GDF15. CCL23 is secreted by senescent T cells, while VEGF and GDF15 are secreted by senescent fibroblasts. **(B)** Metastasis is promoted by GDF15 and CCL5, which are secreted by senescent fibroblasts and T cells, respectively. Senescent T cells contribute to tumorigenesis by inducing inflammation *via* the release of TNF-α. Metastasis is also induced in tumour cells that express the receptor CXCR3 *via* the SASP component CXCL11 secreted by senescent endothelial cells. However, CXCL11 also presents anti-tumorigenic activity by recruiting T cells and NK cells at the site of tumour. **(C)** Tumour apoptosis is induced by IL-29 secreted by senescent T cells. In addition, IL-29 contributes to cancer-specific immune response *via* the recruitment of NK cells. **(D)** Senescent tumour cells evade the immune system *via* the secretion of elevated CXCL12 levels, inducing CXCR4 internalisation in T cells and impairing T cell directional migration.

Fibroblasts represent the major cell type within the connective tissue and are involved in ECM production and maintenance (90). Following fibroblast senescence, SASP components such as growth factors can promote neoplastic alterations that initiate and support metastatic development (**Figure 2**). The ability of senescent fibroblasts to induce tumour cell hyperproliferation has been observed in breast and prostate tumours (91, 92). In regard to colorectal cancer, a study by Guo et al. (2019) showed that senescent fibroblasts accumulate in individuals with advanced adenomas and colon cancer compared to healthy individuals. *In vitro*, senescent fibroblasts promoted the proliferation of both adenoma and colon cancer cells *via* the secretion of the SASP component growth differentiation factor 15 (GDF15) (93). GDF15, also termed macrophage-inhibiting cytokine-1 (MIC-1), is a growth factor belonging to the TGF-β superfamily which has been revealed to accelerate G1-S phase transition, to stimulate angiogenesis and to promote colon cancer metastasis *via* epithelial-to-mesenchymal transition (94–97). Study investigating co-culture of LT97, AA/C1, Caco‐2, and HT‐29 cells with senescent CCD‐18Co fibroblasts determined a 2-fold increase in cell migration and invasion compared to the co-culture with non-senescent CCD‐18Co fibroblasts (p < 0.01) (93). Real-time PCR analysis of the senescent CCD-18Co cells revealed that GDF15 mRNA levels were increased by 10-fold compared to non-senescent CCD-18Co cells (p < 0.05). Western blot analysis of GDF15 in senescent CCD-18Co cells showed that GDF15 protein concentration was increased by 2-fold compared to non-senescent CCD-18Co cells (p < 0.001) (93). In addition, co-culture of LT97, AA/C1, Caco-2 and HT-29 cells with senescent CCD-18Co cells which presented GDF15 knockdown *via* short-hairpin GDF15 (shGDF15) resulted in a 2-fold decrease in cell migration and invasion compared to the controls (p < 0.05) (93).

In support to the hypothesis that T2D, senescence and cancer are related, T2D patients present circulating GDF15 levels that are three times higher compared to the healthy controls (p < 0.001) (98). Although the upregulation of the SASP component GDF15 has been associated with many types of cancers, including colon cancer, its pro-tumorigenic role remains unclear and context-dependent (99). Yang et al. (2020) showed that senescent fibroblasts expressing galactosylceramidase (GALC) enhance the migration ability of CRC cells. Specifically, the co-culture of LoVo cells with GALC-expressing fibroblasts determined a 2-fold increase in LoVo cells migration ability compared to the controls (p < 0.05) (100). Coppé et al. (2006) showed that WI-38 senescent fibroblasts secrete high levels of vascular endothelial growth factor (VEGF) and increase tumour vascularization. The VEGF mRNA and protein levels in senescent WI-38 cells were increased by 2-fold and 5-fold, respectively, compared to the pre-senescent WI-38 cells (p < 0.05). *In vivo*, treatment of tumour-bearing nu/nu mice with senescent fibroblasts determined a 3-fold increase in the number of large blood vessels compared to mice treated with pre-senescent fibroblasts (p < 0.05) (101).

### Endothelial cells

Research concerning the effect of endothelial senescence on cancer has been scarce. Nevertheless, there is some evidence that endothelial senescence promotes tumour growth and metastasis. Borovski et al. (2013) showed that senescent tumour microvascular endothelial cells (tMVEC) favour the growth of glioblastoma (GBM) cells (102). Specifically, co-culture of GBM cells with senescent tMVEC resulted in the 10-fold increase in GBM cell number compared to the negative control (p < 0.05) (102). Hwang et al. (2020) showed that senescent human umbilical vein endothelial cells (HUVEC) secrete the chemokine CXCL11, which promotes breast tumour migration both *in vitro* and *in vivo* (103). Specifically, the co-culture of MDA-MB-231 cells with senescent HUVEC cells transfected with CXCL11 small interfering RNA (siRNA) determined a 10-fold decrease in MDA-MB-231 cell migration compared to the negative control (p < 0.001). *In vivo*, mice xenografts were treated with conditioned medium containing CXCL11 secreted from senescent HUVEC. The tumour volume of MDA-MB-231 cells was increased by 4-fold compared to the volume of MDA-MB-231 cells treated with conditioned medium from senescent HUVEC transfected with CXCL11 siRNA (p < 0.05) (103). Significantly, CXCL11 is involved in CRC growth and metastasis. Gao et al. (2018) showed that CXCL11 downregulation inhibits cell growth and invasion in CRC. Specifically, the cell growth of SW480 cells transfected with CXCL11 siRNA was reduced by 6-fold compared to the negative control (p < 0.01). In addition, the migration of SW480 cells transfected with CXCL11 siRNA was reduced by 5-fold compared to the negative control (p < 0.01) (104). CXCL11 is among the ligands that activate the CXCR3 receptor, which is mainly expressed on effector T cells and NK cells and promotes infiltration into an inflammatory site (105). Although CXCL11 has anti-tumour activity *via* the recruitment of innate and adaptive immune cells at the site of tumour, some colorectal tumours express CXCR3 receptors that function as a metastatic mediator (106). Kawada et al. (2007) showed that CXCR3 activation on Colo205 cells resulted in approximately a 2-fold increase in cell migration compared to the controls (p < 0.01) (107). In addition, Cambien et al. (2009) showed that the treatment of C26 cells with CXCL11 and the CXCR3 inhibitor AMG487 resulted in almost a 2-fold decrease in cell migration compared to the negative control (p < 0.001) (108). Therefore, CRC is plausibly affected by endothelial senescence (**Figure 2**).

### T lymphocytes

Similar to endothelial cells, the effect of T cell senescence on CRC has been scarcely investigated. In support of the idea that T2D-induced T cell senescence is implicated in cancer, Broadway et al. (2021) suggested that T2D-associated T cell senescence has a potential tumorigenic role in ovarian cancer metastasis (109). Because the intestine contains the largest number of immune cells in the human body, including T cells, it is probable that T cell senescence also affects this anatomical location (110). The presence of senescent cells such as EMRA T cells in the TME can contribute to the generation of a pro-inflammatory environment that supports tumorigenesis, therefore contrasting the action of non-senescent tumour-infiltrating cells (TILs) against the tumour as a part of the normal host’s immunity (111). EMRA CD4^+^ and CD8^+^ cells, which have been found to be elevated in T2D patients, possess a unique inflammatory SASP repertoire – proteases, chemokines, interleukins, growth factors and insoluble factors such as extracellular matrix components (29, 43). Particularly, the gene expression of CCL5, CCL23, tumour necrosis factor α (TNF-α) and IL-29 and are upregulated in the EMRA CD8^+^ subset (43). Cambien et al. (2011) showed that CCL5 promotes cell migration and invasion in colon cancer: *in vitro* experiments showed that treatment of CT26 and HT29 cells with 50 ng/ml of CCL5 determined a 2-fold increase in cell proliferation compared to the controls treated with base medium only (p < 0.01). *In vivo*, treatment of CT26-inoculated mice with anti-CCL5 resulted in the 2-fold decrease in tumour incidence (p <0.05) (112). Hwang et al. (2005) showed that CCL23 has pro-tumorigenic potential *via* induction of angiogenesis. Specifically, in chick chorioallantoic membrane (CAM) assay, injection of 10 ng per egg of CCL23 determined a 3-fold increase in blood vessels number compared to the negative control (p < 0.01) (113). The role of TNF-α in tumour growth has been controversial: Carswell et al. (1975) showed that TNF-α was capable of inducing tumour necrosis (114). However, over the years multiple studies demonstrated the pro-tumorigenic activity of TNF-α *via* inflammation (115). For instance, treatment of HCT-116 cells with 20 μg/L of TNF-α determined a 2-fold increase in cell number compared to the control (p = 0.001) (115). In addition, the IL-6 and IL-8 levels in HT-29 cells treated with 5ng/ml of TNF-α were approximately 3 times and 5 times higher, respectively, compared to the controls (p < 0.05) (116).

By contrast, other SASPs such as IL-29 have tumour inhibitory effects *via* the induction of caspase-mediated apoptosis and increase in NK cell activity (117). Specifically, Sato et al. (2006) showed that the caspase activity of B16/F0 cells transfected with IL-29 was doubled compared to both the control and transfected B16/F0 cells treated with the caspase inhibitor Z-VAD-fmk (p < 0.05). In addition, following hepatic injection with IL-29 in two independent experiments, the CD3^-^ NK1.1^+^ cell number increased from 10.9% to 26.9%, whereas the CD3^+^ NK1.1^+^ cell number increased from 1.0% to 11.0% (117). Therefore, the overall outcome of senescent T cell activity on TME depends on the type and amount of SASP released. At present, however, the degree of infiltration of senescent T cells and their influence on the tumour microenvironment is still unclear (118, 119).

The tumorigenic action of EMRA T cells is not limited to the colon/rectum. In fact, the enhanced expression of the protease ADAM28 and the receptor CX3CR1 has the potential to alter the migration of these cells to other tissues (43). Shimoda et al. (2007) showed that ADAM28 binds to the P-selectin glycoprotein ligand-1 (PSGL-1) and enhances PSGL-1/P-selectin-mediated cell adhesion to endothelial cells *in vitro*. Specifically, immunolocalization of HL-60 cells with the recombinant ADAM28 protein rpro-ADAM28s determined the 3-fold increase in P-selectin binding compared to the control (p < 0.01) (120). As a result, the ability of these cells to adhere to the endothelial wall in the absence of stimulation increases the possibility of cell migration to other tissues. CX3CR1 expression in EMRA T cells is three times higher than the control (p < 0.001) (43). CX3CR1 allows cell adhesion to fractalkine-expressing endothelial cells: in two independent experiments, Imai et al. (1997) showed that expression of CX3CR1 in K562 cells determined a 5-fold increase in adhesion to fractalkine-expressing ECV304 endothelial cells compared to the control (121).

### Tumour cells

Cellular senescence is not restricted to only healthy cells but has been observed in several tumour cells: primary neoplastic cells from different types of cancer, including colon cancer, appear to be senescent *in-vitro* and express high levels of SA-β-gal (122, 123). Choi et al. (2021) showed that senescent colorectal cancer cells generate a cytokine shield through their SASP that inhibits intratumoral CD8^+^ T cell infiltration. This is achieved *via* the secretion of high CXCL12 concentration, which induces internalisation of the CXCR4 receptor and results in impaired directional migration (124). Specifically, the CXCL12 mRNA levels in senescent SW480 cells were increased by 6-fold compared to the control (p < 0.01). When CD8^+^ T cells were co-cultured with senescent SW480 cells overexpressing CXCL12, the number of migrated T cells was reduced by almost 2-fold compared to the CD8^+^ T cells cultured with non-senescent SW480 cells (p <0.001). In order to demonstrate that CXCL12 causes downregulation of CXCR4 expression on the plasma membrane, Jurkat cells were treated with 1 mg/mL of recombinant human CXCL12 (rhCXCL12) for 30 minutes and then analysed *via* fluorescence-activated cell sorting (FACS). As a result, the number of CXCR4-expressing cells was reduced from 10^4^-10^5^ cells to 10^3^-10^4^ cells (124). Therefore, tumour senescence represents an additional ability of tumour cells to evade and suppress the host’s immune response (125) (**Figure 2**).

## Detection and therapies

As previously mentioned, CRC still remains incurable and highly lethal in the advanced stage. Thus, improvements in therapies are required to reduce the disease burden. In this section, we will discuss the limitations regarding the traditional chemotherapy and will review some of the recent therapeutic approaches that have shown promising outcomes in targeting both senescent and cancer cells.

### Current chemotherapy and its challenges

The use of conventional CRC chemotherapeutic drugs such as leucovorin and 5-Fluorouracil (5-FU) poses multiple problems in that a patient can develop severe side-effects such as nausea, alopecia, diarrhoea and neutropenia (126, 127). In addition, cytotoxic drugs that provide a cure to metastasis are effective in only a few types of tumours. In tumours such as colorectal, gastric, ovarian and breast cancer, chemotherapy limits to prolong patient’s survival but does not provide a definitive cure. The phenomenon of tumour drug resistance is also observed, suggesting the emergence of mechanisms that counteract the cytotoxic drugs action, leading to the lack of tumour cells sensitivity despite optimal exposure to the drugs (128, 129). An additional effect of chemotherapy is the induction of senescence on the tumour and surrounding cells, a phenomenon referred to as chemotherapy-induced senescence or therapy-induced senescence (TIS) (130, 131). Although TIS is beneficial because it restricts tumour growth, it also has negative consequences. Firstly, the SASP released by senescent tumour cells support tumour growth in the TME, as discussed before. Secondly, senescent tumour cells can escape the senescent state, increasing the risk of tumour relapse (131). Importantly, tumour cells that escape senescence manifest an increased malignancy, drug resistance and a stem-like phenotype. This increased malignancy seems to be associated with the activation of Wnt signalling as a result of TIS (131, 132). As a consequence, while TIS gives an advantage in restricting tumour growth, this advantage is limited to a short period. Therefore, improvements in cancer therapeutic approach are required.

### Senolytic therapy

Chronic senescent cells and tumour senescent cells have great potential to favour tumour growth and metastasis. Senescent cells can be targeted and killed by senolytic drugs. There are several types of senolytics and some of them have been shown to decrease the number of senescent cells in human clinical trials (133). For this reason, senolytics represent a promising therapeutic approach. However, because senescent cells possess a large variety of phenotypes, it has been challenging to find a biomarker that is consistent across cellular senescence in all organ systems (134). One potential biomarker for senescence as well as senolytic drug target is SA-β-Gal (135, 136). Cai et al. (2020) developed the prodrug “senescence-specific killing compound 1” (SSK1) that is specifically cleaved by lysosomal β-gal into cytotoxic gemcitabine (137). SSK1 effectively induced apoptosis and cleared senescent cells in different tissues. Specifically, SSK1 reduced the viability of senescent human umbilical vein endothelial cells (HUVECs) and mouse lung fibroblasts by 2-fold and 8-fold, respectively, compared to the non-senescent controls (p < 0.0001) (137). Importantly, SSK1 eliminated mouse and human senescent cells independently of the senescence inducers. In fact, compared to non-senescent controls, SSK1 determined a 2-fold decrease in the viability of senescent human oesophageal fibroblasts in which senescence has been induced by replication, etoposide, H_2_O_2_, and oncogene-induced senescence (p < 0.0001) (137). In addition, SSK1 determined at least the 2-fold decrease in serum levels of SASP such as IL-6 and CXCL1 in mice (p < 0.01). Therefore, SSK1 has the ability to attenuate SASP-associated inflammation (137). Despite the promising results, currently there are no potent and specific markers of senescent cells (138). In fact, the staining method for SA-β-Gal detection proposed by Dimri et al. (1995) presents some limitations due to its time consumption and lack of sensitivity (27, 139). Cahu et al. (2013) proposed a faster and more sensitive senescence detection method based on flow cytometry. This technique is based on the detection of green fluorescence emitted from the hydrolysis of the molecule 5-dodecanoylaminofluorescein di-β-D-galactopyranoside (C12FDG) by the SA-β-Gal (140).

Another potential biomarker characterising both colorectal cancer cells and senescent cells is the mitochondrial enzyme glutaminase 1 (GLS1) which is involved in the conversion of glutamine into glutamic acid (141, 142). Importantly, cancer cells depend on glutamine metabolism for metabolic and anabolic processes – glutamic acid is involved in ATP production and biosynthesis of amino acids, nucleotides and lipids *via* the conversion of glutamate to α-ketoglutarate (143, 144). Senolytics that inhibit GLS1 represent an alternative therapeutic approach compared to the SA-β-Gal-associated senolytics. Huang et al. (2014) showed that expression of GLS1 is increased in human colorectal cancer tissues and that GLS1 inhibition decreases tumour growth rate, suggesting that GLS1 may be associated with the progression of colorectal cancer. Specifically, GLS1 was strongly expressed in approximately 54% of T3 colorectal tumour tissues (p < 0.001, N = 128) and approximately 46% of T4 colorectal tumour tissues (p < 0.001, N = 78). On the other hand, GLS1 was strongly expressed only in approximately 23% of T1/T2 colorectal tumour tissues (p < 0.001, N = 31). After treatment with the GLS1 inhibitor 6-diazo-5-oxo-L-norleucine (DON) for 48 hours, the growth rate of HT29 cells was reduced by 2-fold (p < 0.0001) (142).

Since the current chemotherapy for cancer is responsible for TIS in several cases, it has been reasoned that senolytics should be used to avoid tumour relapse and to reduce the undesired tumour-promoting effects deriving from the SASP. This led to the proposal of the one-two punch therapeutic strategy for cancer: a first compound is used to induce senescence in cancer cells followed by the use of senolytic drugs to specifically kill those senescent cancer cells (131). However, it is necessary to acknowledge that TIS is not limited to tumour cells: TIS in non-malignant cells has been associated with dysfunction of the heart, kidneys, bone, bone marrow and nervous system, which contribute to the adverse effects of cancer therapy (132). Therefore, while the adoption of senolytics in cancer therapy carries potential promise, there are still several concerns regarding the lack of senolytic action universality, the potential for systemic toxicity, senolytic drug resistance and damage to healthy senescent cells like those contributing to wound-healing (132).

### CAR T cell immunotherapy

**Figure 3:**
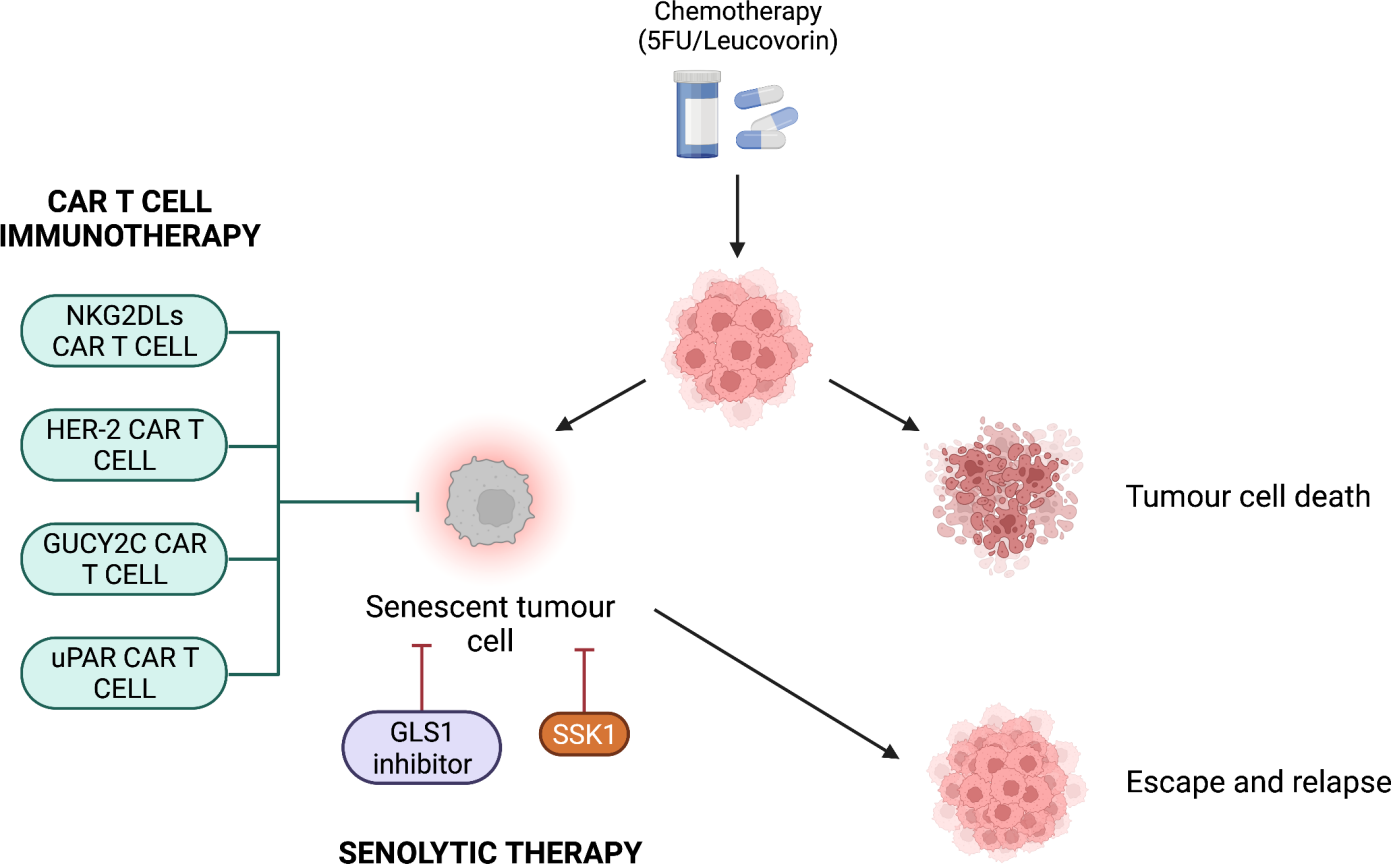
Potential combination therapies for colorectal cancer. After chemotherapeutic treatment, tumour cells are either killed or become senescent. CAR T cell immunotherapy or senolytic therapy can be used to avoid escape from the senescent state and tumour relapse. CAR T cell immunotherapy targets antigens present on colorectal cancer cells such as NKG2DLs, HER-2, GUCY2C and uPAR. Senolytic therapy targets the SA-β-Gal *via* the compound SSK1 or inhibits the mitochondrial enzyme GLS1, which is important for tumour cell metabolism.

Cancer immunotherapy is an innovative therapeutic approach that modulates the host’s immune system to specifically target cancer cells (145). Immunotherapy is particularly important considering that cancer cells use several mechanisms to evade immune surveillance such as downregulation of MHC molecules, Fas ligand-induced apoptosis and upregulation of immune checkpoint molecules (146–148). As such, CAR T cell therapy has been in development to target tumours in various settings with successes (149–151), including in CRC (149, 152–156) (**Figure 3**).

A novel therapeutic approach has been proposed by Amor et al. (2020), who suggested to use CAR T cells as a senolytic therapy. These CAR T cells have been engineered to target the receptor urokinase-type plasminogen activator receptor (uPAR) (157) (**Figure 3**). uPAR is a regulator of ECM proteolysis and it is upregulated in senescence as well as many human cancers (157, 158). In two independent experiments, uPAR-specific CAR T cells completely lysed uPAR-expressing NALM6 cells when the E:T ratio was 1:1. By contrast, with the same E:T ratio, the negative control lysed less than 20% of uPAR-expressing NALM6 cells. In addition, uPAR-specific CAR T cells lysed 80% of senescent KP cells when the E:T ratio was 25:1 (157). *In vivo*, senescence was induced in hepatocytes of immunodeficient NSG mice *via* oncogene-induced senescence, detected through bioluminescence imaging. The treatment with uPAR-specific CAR T cells resulted in the 3-fold decrease in bioluminescence signal compared to the negative control (p = 0.0182). This suggests that uPAR-specific CAR T cells effectively cleared the senescent hepatocytes in NSG mice. Importantly, in support to the one-two punch therapeutic strategy, the authors evaluated the combination of TIS and CAR T cell therapy. Mice with KP lung adenocarcinomas were treated with a combination of MEK and CDK4/6 inhibitors with the purpose to induce KP cell senescence. Treatment for 7 days with uPAR-specific CAR T cells determined a 2-fold increase in the number of infiltrated CD69^+^ CD45.1^+^ CD8^+^ T cells compared to the control (p = 0.0021). This suggests that CAR T cell treatment enhances activated CD8^+^ T cell infiltration within a senescent tumour (157). Thus, CAR T cell immunotherapy against CRC showed favourable results both *in vitro* and *in vivo*, representing a potential candidate for combination therapy.

## Future directions

Further research assessing the efficacy and cost-effectiveness of combination therapy with senolytics and/or CAR T cells is required. These therapies are preferable compared to those focussing exclusively on tumour senescence induction: although Wang et al. (2018) showed that treatment of HCT116 cells with 40 μM of the compound Baicalin reduced the colony survival by 5 fold (p < 0.001), senescent cells have been shown to be resistant to apoptosis (159–161). As shown in **Figure 3**, chemotherapy-treated tumour cells could become senescent and that some could escape and relapse. Moreover, there is a risk that these cells could become resistant to apoptosis, conferring senescent tumour cells a pro-survival advantage and therefore could promote tumour resistance to therapy. Therefore, it may be a better approach to utilise senolytics as a therapeutic strategy rather than tumour senescence induction. However, further investigation is needed to confirm this.

Further research should also assess the use of metformin to treat CRC patients with T2D. Metformin has been associated with decreased risk of CRC in diabetic patients and prognosis improvement in CRC patients with diabetes (162). For instance, Tarhini et al. (2020) showed that the use of metformin in patients with both CRC and T2D is associated with improved overall survival (adjusted hazard ratios [aHR] = 0.45, 95% CI = 0.21 - 0.96) and disease-free survival (aHR = 0.31; 95% CI = 0.18 - 0.54) (163). Furthermore, metformin can be considered as a senolytic drug: metformin treatment has been associated with increased tumour cell apoptosis and inhibition of SASP secretion (164–166). Thus, should metformin be considered as a therapeutic choice in patient with CRC and T2D?

Another area to explore is CRC immunology. Previously, we showed that tumour infiltrating T-, B- and IgA+ plasma cells play key roles in rectal cancer tumour microenvironment (19). For instance, CD20^+^ TIL-B and IgA^+^ cells demonstrated significant associations with long-term survival of patients with rectal cancer. Although we did not investigate T2D, in the future, it would be important to understand whether these immune cells are present in the tumour microenvironment of CRC patients with T2D. Additionally, Saito et al. (2020) reported that CRC and T2D patients treated with metformin induced structural changes and immune cell profile in the tumour microenvironment. It has been shown that metformin increases the immune cell (CD3^+^CD8^+^) infiltration and reduces the rate of M2‐type tissue associated macrophages, and promotes stromal fibrosis in human CRC, which may result in an immunocompetent microenvironment from an immunosuppressive one (167). Thus, along with its senolytic activity, metformin could be utilised as a pro-immunogenic anti-tumour agent.

## Conclusions

Cellular senescence represents a risk factor for colorectal cancer development in type 2 diabetic patients. The pathophysiological events occurring in type 2 diabetes contribute to the generation of premature cellular senescence. Research shows that senescent fibroblasts, endothelial cells and T cells release proinflammatory SASP that favours tumorigenesis, tumour growth and metastasis. However, some SASP have tumour inhibitory effect and therefore the overall outcome of senescence activity on the tumour microenvironment depends on the type and amount of SASP released. Senescence also occurs in tumour cells and enhances the ability of colorectal cancer cells to suppress and evade the host’s immune response. Because conventional chemotherapy increases the risk of tumour senescence and relapse, new therapeutic approaches are required. Senolytic drugs showed favourable results in their ability to clear senescent cells, reducing the likelihood of tumour relapse and cancer immune resistance. In addition, CAR T cell immunotherapy against colorectal cancer showed promising outcomes in their tumour-killing ability both *in vitro* and *in vivo*. Thus, senolytic compounds and CAR T cells represent a potential candidate for combination therapy. Further research with the purpose to expand the knowledge in the field of senescence and cancer is required, allowing improvements for the diagnosis and treatment of senescence-associated diseases.

## Author contributions

FM, PU and DZ drafted the manuscript. NA contributed with discussions and critical revision of the manuscript. MP, DZ and NA conceptualised the project. MP revised, edited, supervised and administered the project. All authors contributed to the article and approved the submitted version.

## Funding

This work was supported by funding Vetenskaps-rådet (The Swedish Research Council, 2017-04663) and Carl Tryggers Stiftelse (CTS:18:279).

## Conflict of interest

The authors declare that the research was conducted in the absence of any commercial or financial relationships that could be construed as a potential conflict of interest.

## Publisher’s note

All claims expressed in this article are solely those of the authors and do not necessarily represent those of their affiliated organizations, or those of the publisher, the editors and the reviewers. Any product that may be evaluated in this article, or claim that may be made by its manufacturer, is not guaranteed or endorsed by the publisher.
